# Extracting the Globally and Locally Adaptive Backbone of Complex Networks

**DOI:** 10.1371/journal.pone.0100428

**Published:** 2014-06-17

**Authors:** Xiaohang Zhang, Zecong Zhang, Han Zhao, Qi Wang, Ji Zhu

**Affiliations:** 1 School of Economics and Management, Beijing University of Posts and Telecommunications, Beijing, China; 2 Department of Statistics, University of Michigan, Ann Arbor, Michigan, United States of America; Semmelweis University, Hungary

## Abstract

A complex network is a useful tool for representing and analyzing complex systems, such as the world-wide web and transportation systems. However, the growing size of complex networks is becoming an obstacle to the understanding of the topological structure and their characteristics. In this study, a globally and locally adaptive network backbone (GLANB) extraction method is proposed. The GLANB method uses the involvement of links in shortest paths and a statistical hypothesis to evaluate the statistical importance of the links; then it extracts the backbone, based on the statistical importance, from the network by filtering the less important links and preserving the more important links; the result is an extracted subnetwork with fewer links and nodes. The GLANB determines the importance of the links by synthetically considering the topological structure, the weights of the links and the degrees of the nodes. The links that have a small weight but are important from the view of topological structure are not belittled. The GLANB method can be applied to all types of networks regardless of whether they are weighted or unweighted and regardless of whether they are directed or undirected. The experiments on four real networks show that the link importance distribution given by the GLANB method has a bimodal shape, which gives a robust classification of the links; moreover, the GLANB method tends to put the nodes that are identified as the core of the network by the k-shell algorithm into the backbone. This method can help us to understand the structure of the networks better, to determine what links are important for transferring information, and to express the network by a backbone easily.

## Introduction

In recent years, complex networks have been investigated by scholars in many domains. The representation, analysis and modeling in complex network theory bring a new paradigm to research on some complex systems including the Internet, transportation systems, biological systems, and social systems [1]. One of the primary aims of complex network research is to reveal the structural characteristics of complex systems. Many emerging concepts, such as the small-world property [2], scale-free behavior [3], community structure [4], and fractality [5], form the basis of our understanding of complex network structure. Because the scales of networks are becoming larger, a more intuitive and efficient method is required to represent and analyze the complex networks. Reducing a large-scale network to an essential backbone can help to solve the conflicts between the large scale of the complex networks and the understanding of the network structure. The backbone of a network is a core component that is extracted by filtering redundant information from the network and preserving far fewer links and nodes from the original network.

The filtering methods for backbone extraction can be divided into two main categories: global methods and local methods. Some global methods use certain global measures to filter the links, such as the link betweenness-based method [6] and the link weight-based method [7]. These methods apply a global threshold on the weights or the betweenness of links in such a way that only those that exceed the threshold are preserved. These filters have been used in the study of functional networks that connect correlated human brain sites [8], food web resistance as a function of link magnitude [9], and mobile communications networks [7]. The link weight-based method, however, could neglect nodes that have a small strength (The strength of node 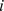 is defined as 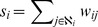, where 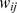 is the weight of the link 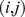 and 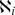 is the set of neighbors of node 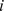) because the introduction of a threshold induces a characteristics scale from the outside [10].

**Figure 1:**
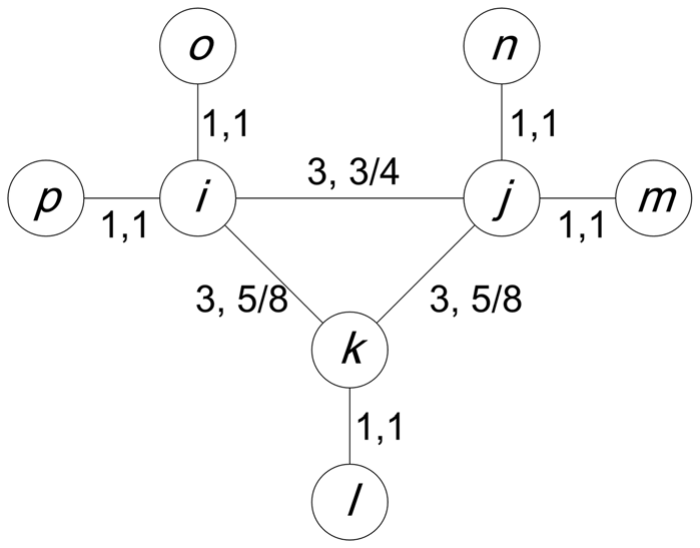
An undirected artificial network. The first number on the line is the value of the link weight, and the second number is the value of the link salience. Although the link 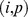 gets the largest value 1 of the link salience, it is only important for node 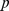. The links 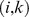 and 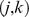 have the smallest value of the link salience, but they are in the core of the network.

The link salience [11], another type of global method, defines the shortest-path tree 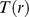 that summarizes the shortest paths from a reference node 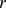 to the remainder of the network and that is conveniently represented by a symmetric 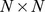 matrix (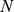 is the number of nodes in the network) that has the element 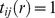 if the link 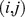 is part of at least one of the shortest paths and 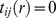 if it is not. The central idea of the approach is based on the notion of the average shortest-path tree that is defined as 
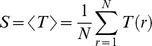
. The element 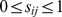 of the matrix 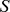 quantifies the fraction of the shortest-path trees that the link 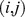 participates in and denotes the salience of the link 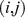. Link salience is a robust approach to classifying network elements because the distribution of 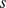, the link salience, exhibits a characteristic bimodal shape on the unit interval in many kinds of networks [11]. Link salience, however, tends to give an higher evaluation to the links being adjacent to low-degree nodes that often lie in the periphery of networks than the links being adjacent to high-degree nodes. For example, in Figure 1, link 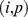 is a part of the shortest-path tree 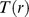 for all of the reference nodes, i.e., 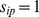, because 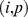 is the only path that connects node 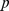 to the remainder of the network. Thus, link 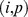 is always a part of the backbone extracted by the link salience method even though the link is meaningful only for node 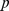
to transfer information between it and the rest of the nodes.

The local methods use local measures to determine which links must be filtered, such as the disparity filter method [10] and the locally adaptive network sparsification (LANS) [12]. The disparity filter method introduces the normalized weight that corresponds to link 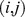 of a certain node 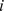 of degree 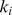 and is defined as 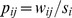, where 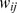 is the weight of the link, 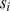 is the strength of node 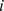. The normalized weight is assumed to be produced by a random assignment from a uniform distribution; thus, the probability density function of 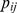 is assumed to be 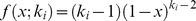. The backbone will include those links whose normalized weights satisfy the relation 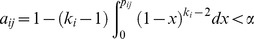 or 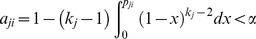, where 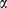 is a specified significance level. Here 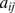 and 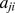 denote significance of the link's normalized weight not following the uniform distribution. The local heterogeneity (Section 3.1) of a link's weight is the premise of the disparity filtering method [10].

**Figure 2:**
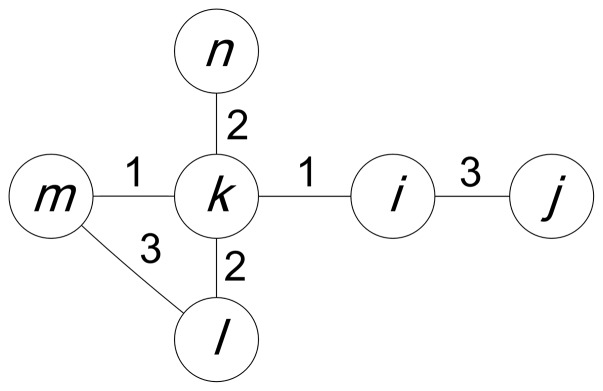
An undirected artificial network. The numbers on the lines denote the weights of the links. Although the weight of link 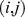 is greater than that of link 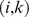, link 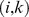 is more important for node 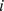 than link 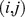 is, because link 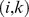 is the only path through which node 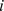 can reach the remainder of the network.

The LANS method, for each node 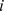 and for any of its neighbors 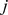, considers the fraction of non-zero links whose weights are less than or equal to 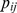, 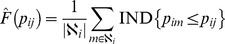, where 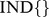 is the indicator function, 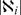 is the number of neighbors of node 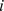, and 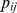 is the normalized weight of link 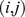. If 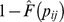 is less than a predetermined significance level 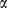, the link 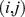 is locally significant and is included in the backbone network. Although both of the local methods do not belittle some links that have small weights from a global view by considering the importance of the links in each specific node, we argue that they could ignore some links that have small weights with respect to the topological aspect. They assume that, for a certain node, its neighboring links (the links that connect to the node) with larger weights are more important. In many cases, however, local and global topological structures of a link determine how important the link is. For example, in Figure 2, although the weight of link 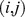 is greater than that of link 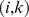, link 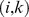 is more important than link 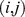 for node 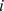 because 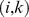 is the path through which 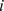 can reach most of the other nodes. From the prospective of information transfer, link 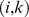 can help node 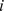 send or receive information more effectively than link 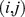 can, because deleting link 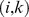 could cause more damage than deleting link 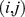 for the information transfer of the network.

Because the local and global methods have advantages and disadvantages, in this study we aim to design a backbone extraction method that accounts for both the global and local topological structure of the networks. And the importance of links is synthetically determined by the weights of the links, the degree of the nodes, and the topological structure. The results of experiments on some real networks show that our propose method has some good characteristics.

## Materials and Methods

In this study, we are inspired by the ideas of link salience and the disparity filter to propose a globally and locally adaptive network backbone (GLANB) extraction method. First, for each specific node, we compute the involvement of its neighboring links, which measures the fraction of the short paths connecting the node to the remainder of the network, which the links participate in. Second, we use a null hypothesis to determine whether each link is statistically important based on its involvement.

### 2.1 Link Involvement

We first consider a weighted, undirected and connected network. We define the length of the link 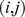 as 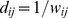, with 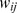 being the weight of link 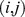, which is consistent with definition of the link length in the link salience method. In most networks the link weights denote the connection strength between nodes. For example, in social networks the link weights often denote the communication frequency between people. Thus, we assume that the links with high weights are important in our case, and we invert the weights to compute the link length that measures the distance between nodes. In practice, the formula of measuring link length should depend on the meaning of the weights. The length of a path that connects two terminal nodes 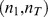 and that consists of 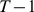 links by a sequence of intermediate nodes 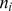, and the link weight 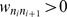 is defined as 
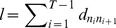
. The shortest path minimizes the total distance 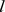 and can be interpreted as the most efficient route between its terminal nodes. The involvement 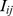 of link 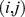 is defined as
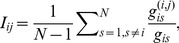
(1)where 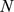 is the number of nodes in the network; 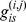 is the number of shortest paths between node 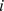 and 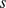 that pass through the link 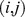; and 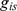 is the total number of shortest paths between node 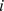 and 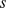. The involvement 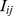 denotes how much the link 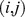 is involved in the most efficient connections between node 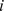 and the other nodes; thus, it can be a measure of the importance of link 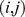 for node 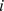 in the view of information transfer between node 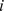 and the remainder of the network. The larger the value of 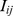 is, the more important link 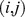 is for node 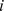. We can see that 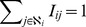, where 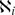 is the set of neighbors of node 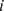.

The involvement is different from the betweenness centrality. The betweenness of link 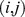 depends on the shortest paths between all pairs of nodes, but the involvement 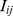 only depends on the shortest paths between the node 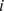 and the rest of the nodes since the definition of involvement 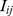 is based on the idea what proportion of the rest of the nodes can connect the node *i* through the link 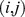. The involvement is also different from the salience because the involvement considers the multiple shortest paths between each pair of nodes, but the salience assumes that only one shortest path exists between a pair of nodes. That is why the GLANB can also be applied to unweighted networks that often have multiple shortest paths between each pair of nodes.

### 2.2 Statistical Importance (*SI*) of Links

We find that the involvement of links that are around a single node is distributed heterogeneously (see Section 3.1). We are interested in the links that have a significant involvement at each given node. However, the local heterogeneity of involvement could simply be produced by random fluctuations. Similar to the disparity filter method, we adopt a null model to compute the random expectation for the distribution of the involvements that is associated with the links of a certain node. The null hypothesis is that the involvement 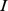 that corresponds to a connection of a certain node of degree 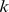 is produced by a random assignment from a probability density function of 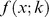. Because the links that are adjacent to a certain node with the degree of 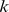 should have the same chance under the random condition to connect the node to the remainder of the network, the mean of the involvement must satisfy the condition
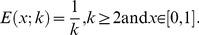
(2)


Many probability density functions satisfy this condition and can be used to generate an involvement that is random and is based on specific assumptions. For example, if we assume that for each specific node that has the degree of 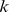, its neighboring links independently participate in the shortest paths between the node and the remainder of the network with a probability of 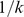; then, the involvement 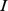 obeys approximately the normal distribution that has a mean of 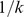 and a variance of 
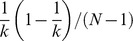
. Alternatively, we can assume that the involvement obeys the power law distribution 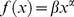 because for most complex networks, the degree and weight have been verified to follow power law distributions [1], [3]. It is easy to obtain the probability density function 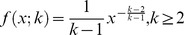. Moreover, the involvement can be assumed to follow a uniform distribution, which is similar to what the disparity filter method has performed for the normalized weights of the links [10] and has the probability density function of 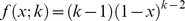.

The GLANB measures the statistical importance 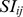 of link 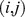 by using a null model to calculate the probability in such a way that its involvement 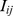 is compatible with the null hypothesis. The statistical importance 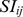 of link 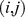 is defined as
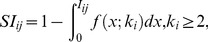
(3)where 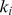 is the degree of node 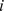. In this study, the involvement is assumed to follow a uniform distribution, i.e., 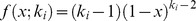; thus, 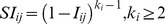. To control the impact of the degree on the statistical importance, we add a parameter 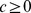 to the formula, as follows:



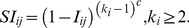
(4)If 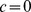, then the statistical importance 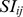 is determined only by 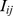 and is not affected directly by the degree (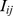 can be affected indirectly by 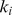 because the shortest paths to node 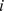 are affected by 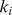). As 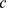 increases, the impact of the degree becomes larger. The experimental results show some interesting characteristics of the GLANB method under different values of 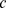 (see Section 3).

The smaller the value of 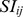 is, the more significantly the link 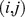 is not compatible with a random distribution; furthermore, the link 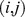 can be considered more important due to the network-organizing principles. The final statistical importance of an undirected link 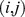 is the minimum of 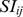 and 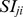. In the case when a node 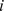 of degree 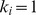 is connected to a node 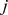 of degree 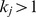, the statistical importance of link 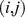 is 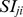. The GLANB can identify a backbone of a network by setting the significance level 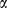 for the 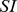 (a link is included in the backbone if its 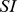 is less than 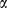) based on the distribution of 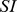 (see Section 3.3), or identify the hierarchical backbones by setting different significance levels since the backbone under high significance level will contain the backbone under low significance level. The backbone includes the links that are statistically important according to the specified significance level and their terminal nodes.

### 2.3 Unweighted and Directed Networks

The GLANB method can be easily applied in unweighted networks. In this case, the weights of all of the links are treated as being equal; thus, the length of a path is the number of links that lie in the path.

To be applied in directed networks, the GLANB must be modified. The directed link 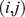 from starting node 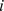 to ending node 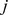 is either an out-link for node 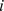 or an in-link for node 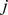. Thus, we define the out (in) involvement 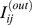 (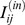) of the directed link 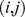 separately as


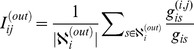
 and



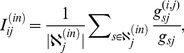
(5)where 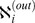 is the set of nodes that can be reached from node 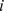 through a directed path, and 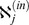 is the set of nodes that can reach node 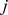 through a directed path; 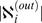 denotes the size of 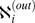; 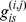 is the number of shortest paths from node 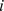 to 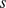 that pass through the link 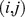; and 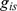 is the total number of shortest paths from node 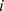 to 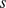. The involvement 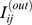 measures how much the link 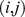 is involved in the shortest paths from node 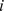 to the other nodes, and 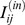 measures how much the link 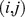 is involved in the shortest paths from the other nodes to node 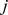.

The statistical importance of link 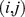 is composed of two parts, the in-importance 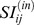 and the out-importance 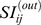, which are defined from the viewpoint of the starting node 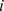 and the ending node 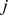 separately as


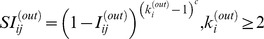
 and



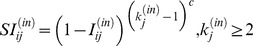
(6)where 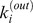 is the out-degree of node 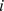, 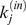 is the in-degree of node 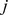, and 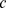 is the control parameter. The final statistical importance of the directed link 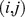 is determined by the minimum of 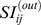 and 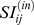. Similar to the case of weighted and undirected networks, the GLANB can identify a backbone from unweighted or directed network by setting a significance level for 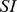 based on the distribution of 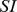, or a hierarchical backbone by setting different significance levels for 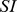.

## Results

To test the performance of the GLANB method, we apply it to four real-world networks, a collaboration network (coauthor) [13], an instant-message network (fetion), an email network (email) [14] and an airport traffic network (airport). We compare the obtained results with those obtained by the disparity filtering method and the link salience method. (1) The collaboration network is based on co-authorship of academic papers in the high-energy physics community from 1995–1999. Nodes represent individuals, and links measure the number of papers that were co-authored. The data are publicly available at http://www-personal.umich.edu/~mejn/netdata/. (2) The instant-message network is based on an instant-message tool, fetion, which is provided by Mobile Corporate. The nodes represent fetion users, and the links measure the number of messages sent between each pair of users. (3) The email network is an undirected and unweighted network. The nodes represent email users, and the links represent whether any communication exists between each pair of users. The email network data are available at http://deim.urv.cat/~aarenas/data/welcome.htm. (4) The airport traffic network is a weighted and directed network. It measures global air traffic that is based on flight data that is provided by OAG Worldwide Ltd. (http://www.oag.com), and it includes all of the scheduled commercial flights in the world in 2011. The nodes represent airports worldwide. The link weights measure the total number of passengers that travel between a pair of airports by direct flights per year. This network is well represented in the literature [15], [16], [17]. In the experiments only the largest connected subnetworks of each of the networks are used. The backbone includes the links that are significantly important according to the extraction methods and their terminal nodes. Because the authors in [11] do not mention how the salience method deals with the directed or unweighted networks, we do not apply the salience method to the email and the airport networks.

### 3.1 Local Heterogeneity of the Link Involvement

The condition under which the null model can perform well is that for each node, its links' involvement shows heterogeneity. If this condition is not satisfied, then it is difficult to identify the important links through the GLANB method. To assess the effect of heterogeneities in the links' involvements at the local level, for each node 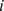 of degree 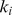, one can calculate the function [18], [19]

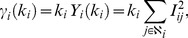
(7)where 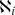 is the set of neighbors of node 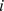 and 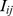 is the involvement of link 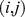.

**Figure 3:**
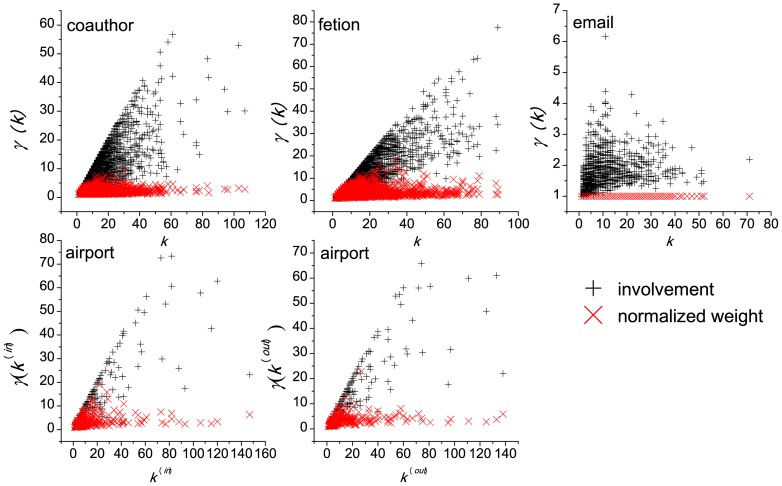
The local heterogeneity of the involvement and the normalized weight in four real networks. Each point in the figure denotes a node in the network. The local heterogeneity of the involvement for node 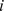 is defined as 
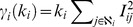
, where 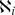 is the set of neighbors of node 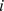, 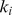 is the degree of node 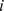, and 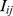 is the involvement of link 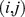. The local heterogeneity of the normalized weight for node 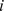 is defined as 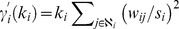, where 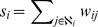 is the strength of node 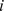. We can find that for all of the networks, the involvement is locally more heterogeneous than the normalized weight is.

As a standard indicator of measuring the concentration of data, the function 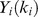 has been extensively used in various domains, including ecology, economics, physics, and complex networks [10], [19], where it is known as the disparity measure. Under perfect homogeneity, when all of the links share the same amount of the involvement of node 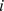
(i.e., 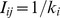), 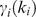 equals 1 independently of 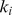, while in the case of perfect heterogeneity, when only one of the links carries the whole involvement of the node, the function is 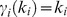. In this way, this function can be used as a preliminary indicator of the presence of local heterogeneity. When local heterogeneity of involvements exists, the GLANB can be more useful than in the case of homogeneity because the GLANB aims to identify the links whose involvements are significantly higher than other neighboring links'. To compare the involvement with the weights of the links, we also compute the heterogeneity of the normalized weights [10] by
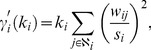
(8)where 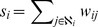 is the strength of node 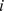. Figure 3 shows the local heterogeneity of the involvement and the normalized weight in the coauthor, fetion, email, and airport networks. We can find that for all of the networks, the involvement is locally more heterogeneous than the normalized weight is (Figure 3). These results indicate that applying the null model to the involvement can identify the statistically important links well.

### 3.2 Size of the Backbones

**Figure 4:**
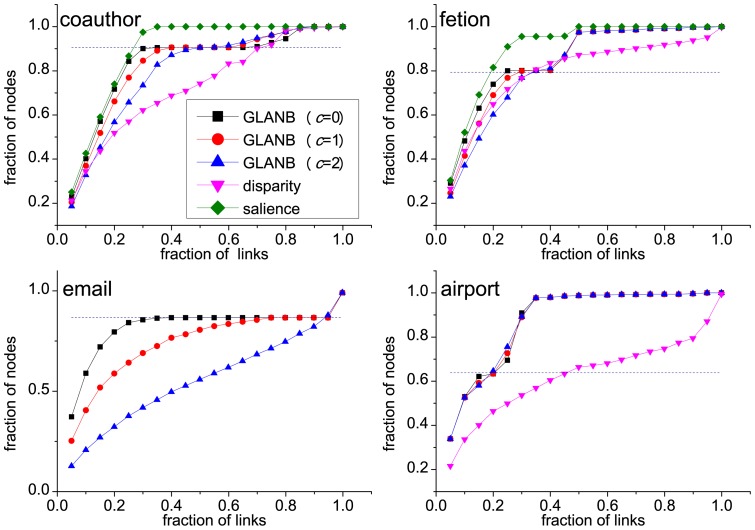
Fraction of nodes maintained in the backbones. The fraction of nodes is a function of the fraction of links retained by the filters. The dash lines correspond to the fraction of the nodes whose degree is greater than 1 in the networks.

The main purpose of extracting backbones is to reduce the number of links in networks, while keeping more nodes. To measure the effects of these filtering methods on the extracted backbones, we analyze the relative sizes of the backbones as a function of the preserved fractions of the links when the network is filtered by the disparity filter, by the link salience and by the GLANB (Figure 4).

For the four real networks, the link salience method can preserve the largest fraction of nodes in the backbone, and the disparity filter method preserves the smallest (except when the fraction of links is less than 0.4 for the fetion network) when the same fraction of links is maintained. The results of the GLANB methods fall in between the disparity and the salience methods. We must note that for the salience method, all of the links that are adjacent to the nodes with a degree of 1 have the largest salience of 1, and preserving these links can retain at least one node. Thus, the link salience method can preserve the largest fraction of the nodes when filtering the networks.

We also find that in the backbone of the coauthor and fetion networks identified by the GLANB method at the specified values of control parameter 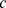, the fraction of nodes stays approximately unchanged for an interval of the fraction of links when the fraction of nodes reaches the threshold that is the fraction of nodes with a degree greater than 1. For the email networks, this phenomenon also exists when 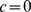 or 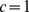. For the airport networks, this phenomenon exists when 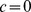. The interval of keeping unchanged is the longest for all of the networks when 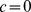 (Figure 4). The reason of the phenomenon is that for the nodes that have a degree of 1, the value of 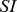 of their neighboring links is very close to 1; thus, these nodes are difficult to include in the backbone when the fraction of links in the backbone is not sufficiently large. Moreover, as the control parameter 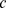 increases, the growth curves of the fraction of nodes become relatively flat (Figure 4), because high value of 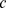 prefers the links that correspond to the nodes that have a high degree, and these links have a low value of 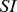. Preserving these links in the backbone cannot increase the fraction of nodes proportionally because some other links that could have been preserved in the backbone are more likely to share the same terminal nodes with them. Thus, these results indicate that the parameter 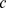 can control the size of extracted backbone by impacting the degrees of the nodes on the value of the involvement.

### 3.3 Robust Classification of Links Based on the Statistical Importance

**Figure 5:**
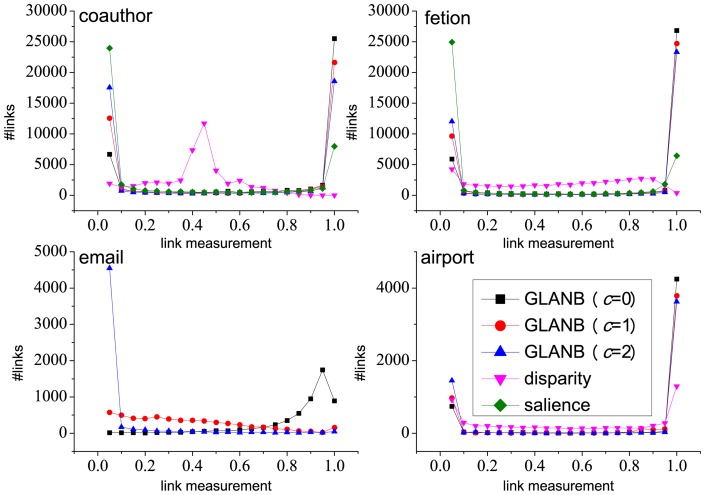
The distributions of the link salience, the link statistical importance and the disparity filtering importance. Link measurement refers to the values of the link salience, link statistical importance, and the disparity filtering importance that are given by the salience, GLANB and disparity methods separately. For the GLANB and disparity methods, the smaller values mean higher importance. For the salience method, the larger values mean higher importance.

Similar to the link salience measure [11], the surprising feature of the statistical importance 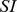 is that the distribution 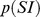 exhibits a characteristic bimodal shape on the unit interval (Figure 5). The networks' links naturally accumulate at the boundaries and have a small fraction at intermediate values. The statistical importance thus successfully classifies network links into two groups: important (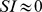) or non-important (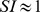). Because a small fraction of links fall into the intermediate range, the resulting classification is not significantly sensitive to an imposed threshold. This circumstance is fundamentally different from some link centrality measures, such as weight and betweenness, which possess broad distributions and which require external and often arbitrary threshold parameters to perform meaningful classifications. The distribution of links' statistical relevance when measured by the disparity filter method shows a unimodal shape in the coauthor network or a flat distribution in the fetion network (Figure 5), which has the result that choosing the appropriate significance level 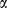 to filter links becomes difficult. For the GLANB method, as the control parameter 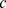 increases, the number of links with high importance increases (Figure 5). The reason is that the GLANB method with a high value of 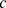 favors the links that correspond to the nodes that have the degree *k*>1, and these links occupy a large proportion of total links.

### 3.4 K-shell distribution of links

To deeply explore the hierarchy of links in the backbones that are extracted by the GLANB, disparity filter and salience methods, we use the k-shell decomposition method to compare the topological distribution of extracted links. The k-shell decomposition method is often used to identify the core and the periphery of the networks [20], [21]. Although the k-shell method only takes into account the nodes' degree not the link weights, it provides a way to compare the backbone extraction methods from the view of topological structure. The process of the k-shell decomposition starts by removing all of the nodes that have one link (degree 1) only, until no more such nodes remain; then, it assigns them to the 1-shell. In the same manner, it recursively removes all of the nodes that have a degree of 2 (or less), creating the 2-shell. This process continues, increasing k until all of the nodes in the network have been assigned to one of the shells. The shells that have high indices lie in the core of the network. To assign all of the links to the shells, we define the shell index of a link as the minimum of its two terminal nodes' shell indices.

**Figure 6:**
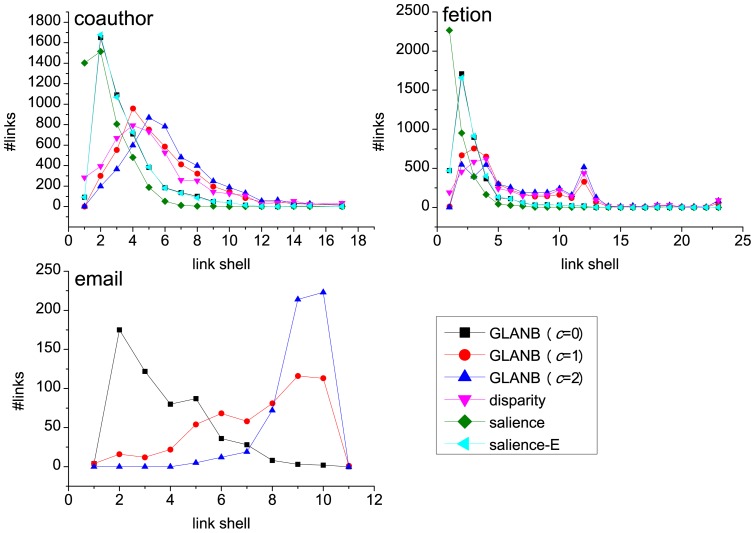
The distribution of links in link-shells. For the coauthor, fetion and email networks, we extract the top 10% important links, based on the GLANB, disparity filter and salience methods separately, to analyze their distributions in terms of link-shells. In addition, we also exclude the links that have degree of 1 to extract the remaining top 10% important links based on the salience method (salience-E) to analyze the distribution.

For the coauthor, fetion and email networks, we extract the top 10% important links based on the 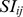 of GLANB (from low to high), the 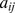 of disparity filter (from low to high) and the 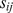 of salience methods (from high to low) separately to analyze their distributions in terms of link-shells. Because the salience method ranks the links for which one terminal node has the degree of 1 as most important, and because both the disparity filter and the GLANB methods rank them as least important, we also exclude these links to extract the remaining top 10% important links based on the salience method (salience-E) to analyze the distribution. The distributions of the links in the range of the shell index are shown in Figure 6. We can see that compared with the disparity and salience methods, the GLANB (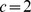) extracts more links that lie in the higher shells, i.e., the topological core of the networks. Especially for the salience method, most of the extracted links lie in the lower shells. This circumstance occurs because the links whose terminal nodes have a low degree tend to have a high salience. For example, the links that are adjacent to the nodes that have the degree of 1 have the highest salience of 1, which means that all of the links in the 1-shell are certain to be in the backbone that is extracted by the link salience method. For the salience-E method, most of the links still fall in the low shells, and the distribution almost coincides with that of the GLANB (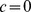), which ignores the degree of the corresponding nodes in a similar way as the salience method. As the control parameter 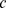 increases, more links fall into the higher link-shells.

There are two reasons to explain why the GLANB (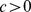) is more likely to extract links from the topological core of the networks than the other methods. One reason is that the backbone which is extracted by the GLANB (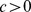) method does not include the links that are adjacent to the nodes that have a degree of 1. The second reason is that the null model depends on the degrees of the nodes. When 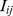 stays unchanged, increasing the value of degree 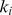 can decrease the value of 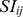 in a power-law way (see formula 4). The larger the value of 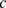 is, the more greatly 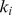 affects 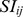. Thus, some links that have a higher shell index would be in the backbone even though their involvement values are not very high. Furthermore, from Figure 3, we can see that the distribution of link involvements for the nodes that have a higher degree shows heterogeneity, which means that some links have both a high-degree terminal and a high involvement.

## Discussion

The GLANB method accounts for both the global and local topology structure of the network when extracting the backbones. On the one hand, the involvement of each link is either a global measure (because it depends on the shortest paths that are determined by the global network structure and the link weights) or a local measure (because the sum of the involvements of the links that are adjacent to any certain node has the value of 1). On the other hand, the null model that is adopted in GLANB is based on a local view because the probability density function depends on the degree of each certain node. Thus, the GLANB determines the importance of the links by synthetically considering the topological structure, the weights of the links and the degrees of the nodes. In this method, the links that have a small weight but are important from the view of structure are not belittled. Furthermore, introducing the control parameter 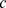 into GLANB provides a way to adjust the impacts of the node degrees on the extracted backbones, which makes the backbone adaptive to the global structure and the local structure by changing the value of 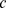. When 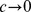, the backbone mainly concentrates on the global structure. When the value of 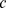 becomes larger, the backbone is affected more greatly by the local structure. Another advantage is that the GLANB method can be applied to all types of networks regardless of whether they are weighted or unweighted and regardless of whether they are directed or undirected.

The computational complexity of the GLANB method is determined by the computation of the involvement and the statistical importance of the links. To compute the involvement, we must find all of the shortest paths between each pair of nodes, which results in the computational complexity being 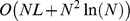 [22], where 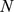 is the number of nodes and 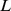
the number of links in the network. The computation of the statistical importance must scan all of the links to compute the degrees of the nodes and the 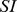 of the links; thus, the computational complexity is 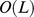. Because 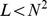, the computational complexity of GLANB is 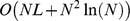. When the size of the network is very large, GLANB is not adaptable if it is executed on only a single computer. However, the computational environment has recently been changing dramatically. Parallel computation platforms are being used pervasively because of their low implementation costs and high performance. Because the GLANB method is based on each single node to measure the involvement and statistical importance of their neighboring links, it is easy to implement GLANB on a parallel platform.

The experiments on the real-world networks show some interesting results. First, the link involvements show local heterogeneity that arises from the topological structure of the networks and from the heterogeneous weight distributions because the shortest paths are determined by those two aspects. Moreover, the involvement is more heterogeneous in the weighted network than in the unweighted network (Figure 3). Second, the link importance distribution, which shows a bimodal shape, gives a robust classification of the links. The bimodal distribution comes from both the local heterogeneity of involvement and the null model that is adopted in GLANB. Third, as the fraction of links in the backbones increases, the size of the backbones that are extracted by the GLANB method first increases rapidly and then becomes almost unchanged, and at last, increases again. The GLANB method assesses the links that are adjacent to the nodes that have a degree of 1 as the least important; thus, as the number of links in the backbone increases, the size of the extracted backbone becomes unchanged for an interval when only the nodes with a degree of 1 are not included in the backbone. Fourth, the control parameter 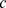 can affect the size of the backbones. A larger value of 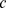 decreases the growth rate of the size of the backbone, because the links that are adjacent to the nodes that have a larger degree are favored, and they cannot efficiently add more nodes into the backbone. Fifth, the GLANB method tends to give more importance to the nodes that are in the core of the network than the other methods do. Especially as the control parameter 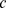 increases, more nodes in the core are included in the backbone. In practice, the choice of 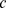 value depends on what backbone is needed. The larger the value of 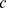 is, more likely the backbone includes the links that are adjacent to nodes with high degree and that are in the core of network from the view of k-shells, and more likely includes less nodes at preserving the same proportion of links.

The GLANB method aims to extract backbones from networks by filtering unimportant links, which can decrease the size of the network greatly. Thus, this method can help us to understand the structure of the networks better, to determine what links are important to transferring information, to express the network by a graph picture easily, and to control the network densities.
